# Evaluation of titanium-prepared platelet-rich fibrin and leucocyte platelet-rich fibrin in the treatment of intra-bony defects: A randomized clinical trial

**DOI:** 10.34172/joddd.2020.020

**Published:** 2020-06-17

**Authors:** Shiva Shankar Gummaluri, Hirak S Bhattacharya, Madhusudan Astekar, Shivani Cheruvu

**Affiliations:** ^1^Department of Periodontology and Implantology, Institute of Dental Sciences, Bareilly, Uttar Pradesh, India; ^2^Department of Oral Pathology and Microbiology, Institute of Dental Sciences, Bareilly, Uttar Pradesh, India; ^3^Department of Periodontology and Implantology, SIBAR Institute of Dental Sciences, Guntur, Andhra Pradesh, India

**Keywords:** Bone regeneration, Chronic periodontitis, Periodontal pocket, Debridement, Platelet-rich fibrin

## Abstract

**Background.** Various treatment modalities, such as leucocyte platelet-rich fibrin (L-PRF), bone grafts, and membranes, have been used for the restoration of lost periodontal tissues. Titanium-prepared platelet-rich fibrin (T-PRF) has attracted attention for its proper haemocompatibility, thick fibrin meshwork, and long resorption time. The present study aimed to evaluate the effectiveness of T-PRF and L-PRF in the management of intra-bony defects based on clinical and radiographic criteria.

**Methods.** Twenty-six subjects with 34 intra-bony 3- walled defects were divided into two groups (n=17) and treated with T-PRF or L-PRF. Clinical and radiographic measurements were recorded at baseline and 6- , 3- and 9- month intervals and tabulated on Microsoft Excel spreadsheets. For intra- and intergroup comparisons, paired and unpaired t-tests were performed. P<0.05 was set as statistically significant

**Results.** Intra-group comparisons revealed statistically significant differences (P<0.05) from baseline in both groups regarding clinical measurements. On intergroup comparison, the T-PRF group exhibited a significantly higher defect fill compared to the L-PRF group (P<0.05).

**Conclusion.** Within the limits of the present study, T-PRF seems to be a better alternative to L-PRF in the treatment of intra-bony defects.

## Introduction

Periodontitis is a multifactorial inflammatory disease destroying the hard and soft tissues of the periodontium.^1^ The goal of any periodontal therapy is to reduce inflammation, decrease probing pocket depth (PPD), and increase clinical attachment level (CAL). Various treatment modalities, such as surgical [(open flap debridement (OFD)] and non-surgical periodontal therapies, have been used in this regard, but the efficacy of treatment is considered only when a therapeutic modality improves osseous lesions.^2^ Although OFD helps reduce the PPD and increase CAL, it cannot regenerate the lost tissues in periodontitis.^3^

Treatment of intrabony defects (IBD) is always a challenging task for a periodontist. For the restoration of this lost bone, various bone graft materials and regenerative techniques have been implemented previously.^4^ Later on, research efforts shifted toward platelet concentrates because of the acceleration of wound healing and stimulation of adjacent cells for the restoration of the lost periodontium.

Platelet concentrates are way ahead of other biomaterials due to constant release of growth factors over a time period, which are crucial for stimulation of adjacent progenitor cells, leading to periodontal regeneration and tissue healing.^5^ These platelet concentrates also have leucocytes, vitronectin, fibronectin, bone morphogenetic proteins (BMPs), and cytokines that contribute to different stages of the wound healing process.^6^Initially, platelet-rich plasma (PRP) was used alone or with bone graft in the treatment of intrabony defects. Although the results were reasonable, due to the risk of antigenicity as a result of the addition of bovine anti-thrombin, leucocyte-platelet-rich fibrin (L-PRF) was introduced, which does not require any kind of anticoagulant.

Since the introduction of L-PRF in 2001 by Choukroun et al,^7^ it has been used extensively in the treatment of intrabony defects and achieved excellent results. Studies by Sharma et al^8^ and Pradeep et al^9^ showed that L-PRF, when used along with OFD, improved the clinical and radiographic parameters. These conclusions gained further grounds by a meta-analysis by Li et al,^10^ where L-PRF improved the parameters significantly.

However, due to current controversies over silica cross-contamination expressed by O’Connell^11^ and Tunali et al,^12^ with the use of glass tubes or glass-coated Vacutainer tubes and shorter resorption time of 7–11 days in L-PRF,^13^ researchers began to search for a better biomaterial. The focus was soon shifted to titanium because of its property of osseointegration, better haemocompatibility, and platelet activation compared to silica. Tunali et al^12^ introduced titanium-prepared platelet-rich fibrin. Titanium is passivated into an oxide layer within itself, which activates platelets and forms a thicker fibrin clot. In addition to the above benefits, T-PRF also has longer resorption time.^14^ Histological studies by Tunali et al^14^ and Chatterjee et al^15^ revealed that T-PRF had thicker fibrin meshwork and higher cellular entrapment, causing more cellularity at the required site and leading to periodontal regeneration.

Since only a limited number of comparative randomized clinical trials have been conducted on T-PRF and L-PRF, the present study aimed to evaluate the effectiveness of T-PRF and L-PRF, based on clinical and radiographic evaluations, in the management of intra-bony defects.

## Methods

### Sample size calculation 

The sample size was calculated using a G-Power software 3.1. A sample size of 34 was obtained when the study power was set at 80% with an effect size of 0.25 and an alpha value of 5%.

### Study design, patient selection, and recruitment

**Figure 1:**
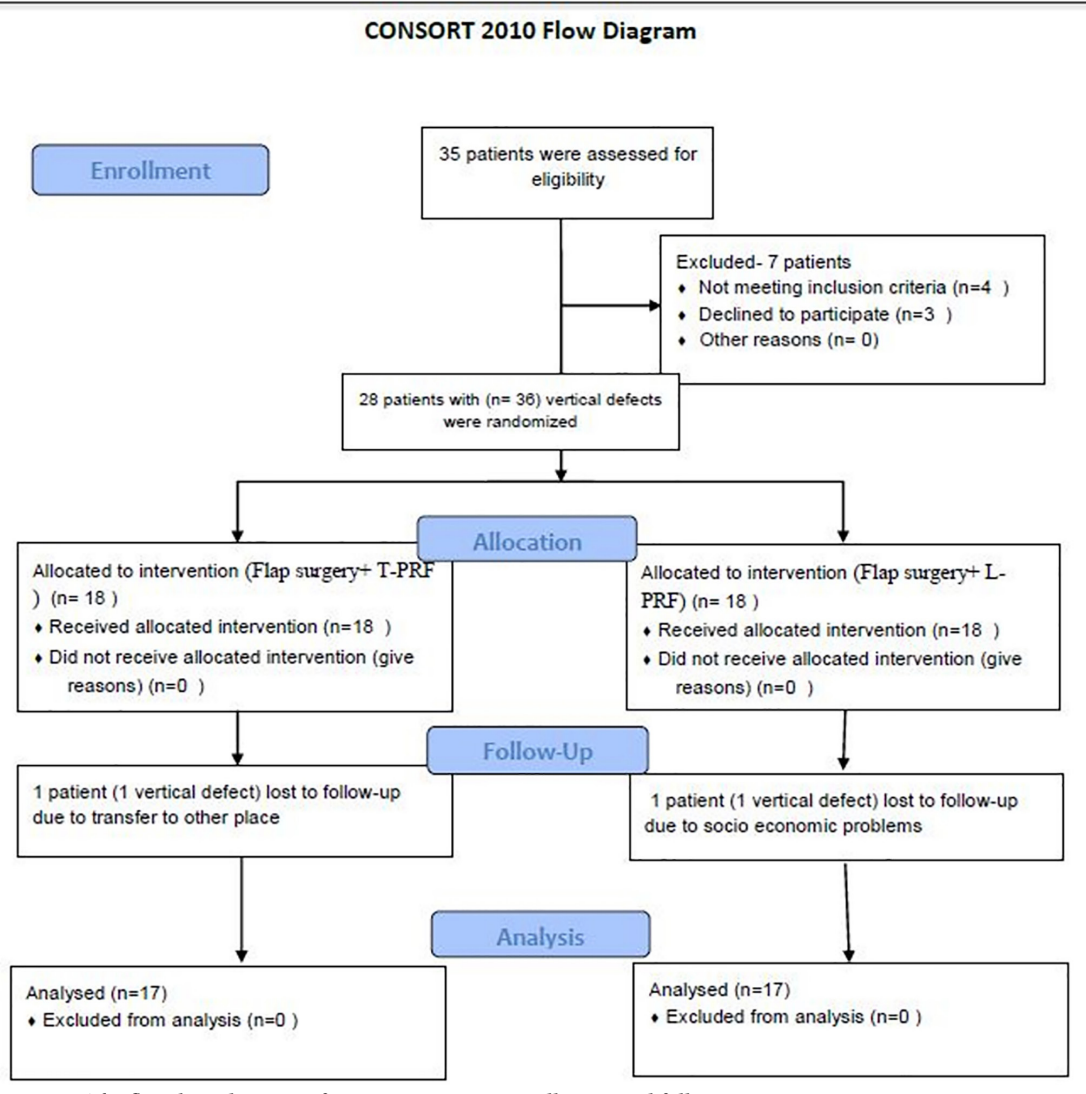
The flowchart diagram of patient screening, enrollment, and follow-up.

The present prospective, single-blind, randomized clinical trial screened 35 patients from the out-patient Department of Periodontics (Figure 1). Before the surgical interventions, approval was obtained from the Institutional Ethics Committee (IDS/ETHCC/17/08). The patients submitted their written informed consent and were selected based on the inclusion criteria. The trial was executed according to the Helsinki Declaration of 1975, modified in 2008. Of 35 patients, 28 patients (17 males and 11 females) had 36 three-walled IBDs. The patients were recruited from July 2018 to May 2019. After recruitment, the patients were randomly assigned to two groups based on the toss of a coin. All the surgeries were performed by one operator (GSS), whereas clinical and radiographic measurements were recorded by another operator (HB) without any knowledge about the groups. The patients were aware that they were going to receive periodontal therapy, but they were blinded to which group they belonged. During the recall visits, two patients (one in each group) lost the follow-up. One patient lost it due to being transferred to a different area and the other because of his socioeconomic issues.

### Inclusion criteria 

The following patients were included in the study: Patients having a minimum of 20 teeth, an age range of 20–55 years, clinical attachment loss of ≥3 mm with probing pocket depth of ≥5 mm in more than two teeth (based on the new system for staging and grading periodontitis in 2017),^16^ confirmation of at least one favorable intrabony defect of ≥3 mm radiographically and re-evaluation of patients who had plaque and gingival indices (GI and PI) of ≤1.

### Exclusion criteria

The following patients were excluded from the study: Patients with systemic diseases, taking medications affecting wound healing and periodontal therapy, reduced platelet counts (<2,00,000/mL), pregnancy, lactation, tooth mobility, smoking, a history of periodontal surgery within one year.

### Clinical parameters assessment 

**Figure 2:**
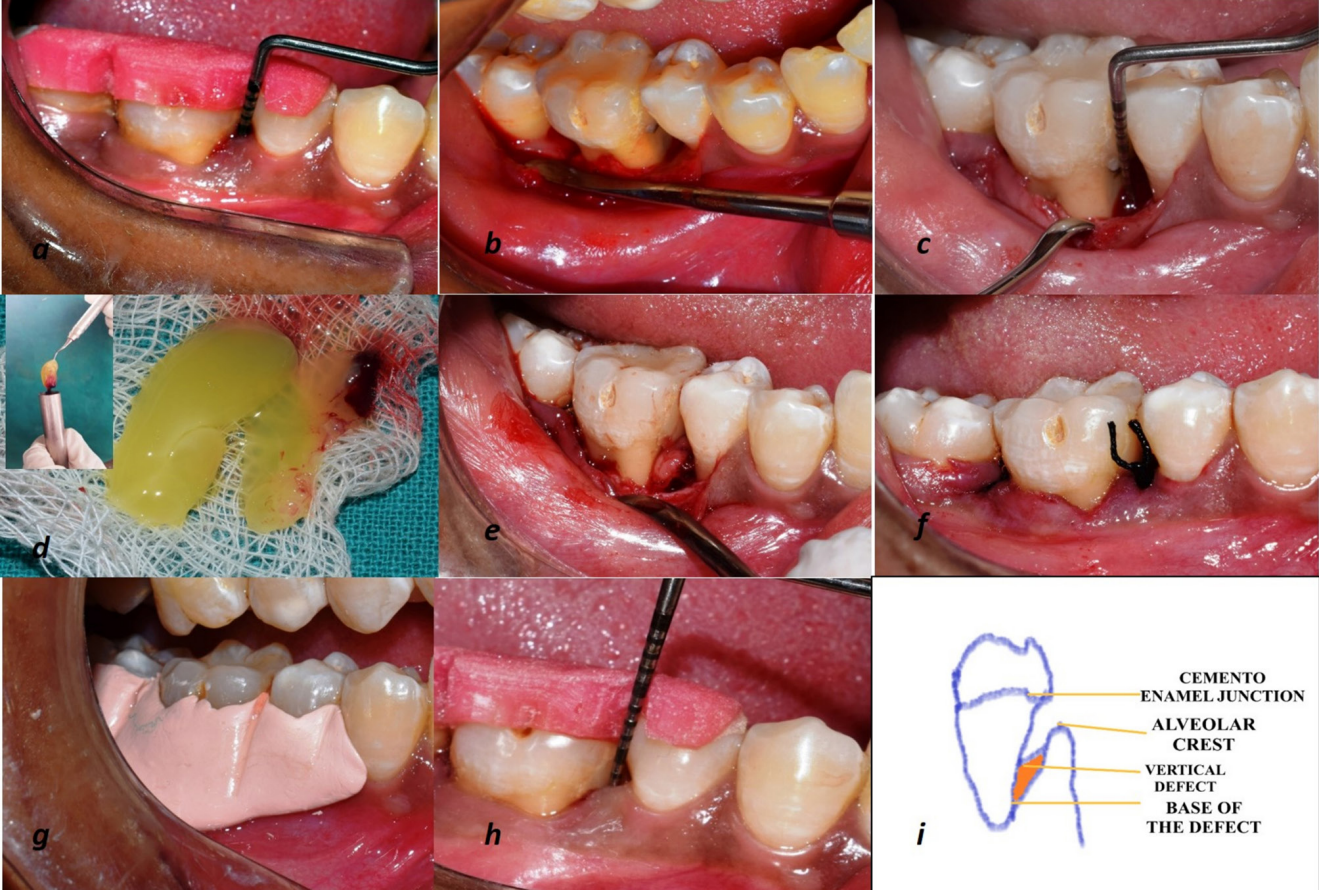
The surgical images of T-PRF group, which includes preoperative probing depth (a), flap reflection and debridement (b), surgical site showing intrabony defect (c), T-PRF membrane (d), T-PRF membrane placed at defect site (e), suturing (f), COE-PAK placed (g), 9 months post-operative probing depth (h) and (i) depicts the pictorial representation of Vertical Defect.

Plaque index (PI),^17^ gingival index (GI),^18^ probing pocket depth (PPD), and clinical attachment level (CAL) were recorded at baseline, and 3, 6, and 9 months postoperatively using a University North Carolina (UNC)-15 probe (Hu-Friedy, USA) (Figure 2). For accurate measurements, acrylic stents with grooves were customized for every patient.^19^ Intra-examiner calibration was achieved when 10 patients’ measurements were assessed 24 hours apart before the initiation of the study. Calibration was taken into account only when the values at baseline and 24 hours were similar up to ±1 mm at 90% level.^20^

### Radiographic parameters assessment 

**Figure 3:**
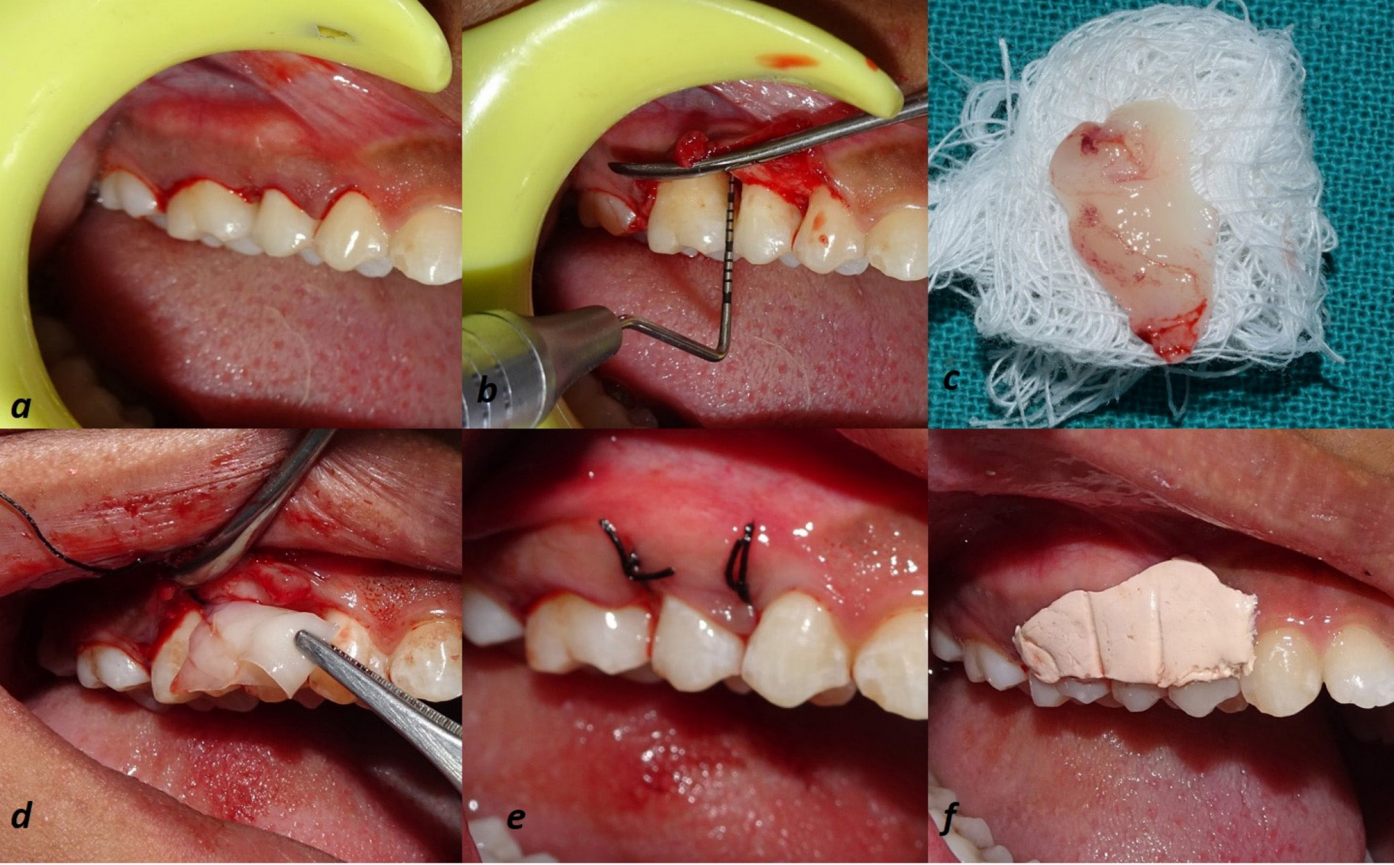
The surgical images of the L-PRF group, which include incision (a), flap reflection and debridement (b), L-PRF membrane (c), L-PRF membrane placement at defect site (d), suturing (e), after placing COE-PAK (f).

**Figure 4:**
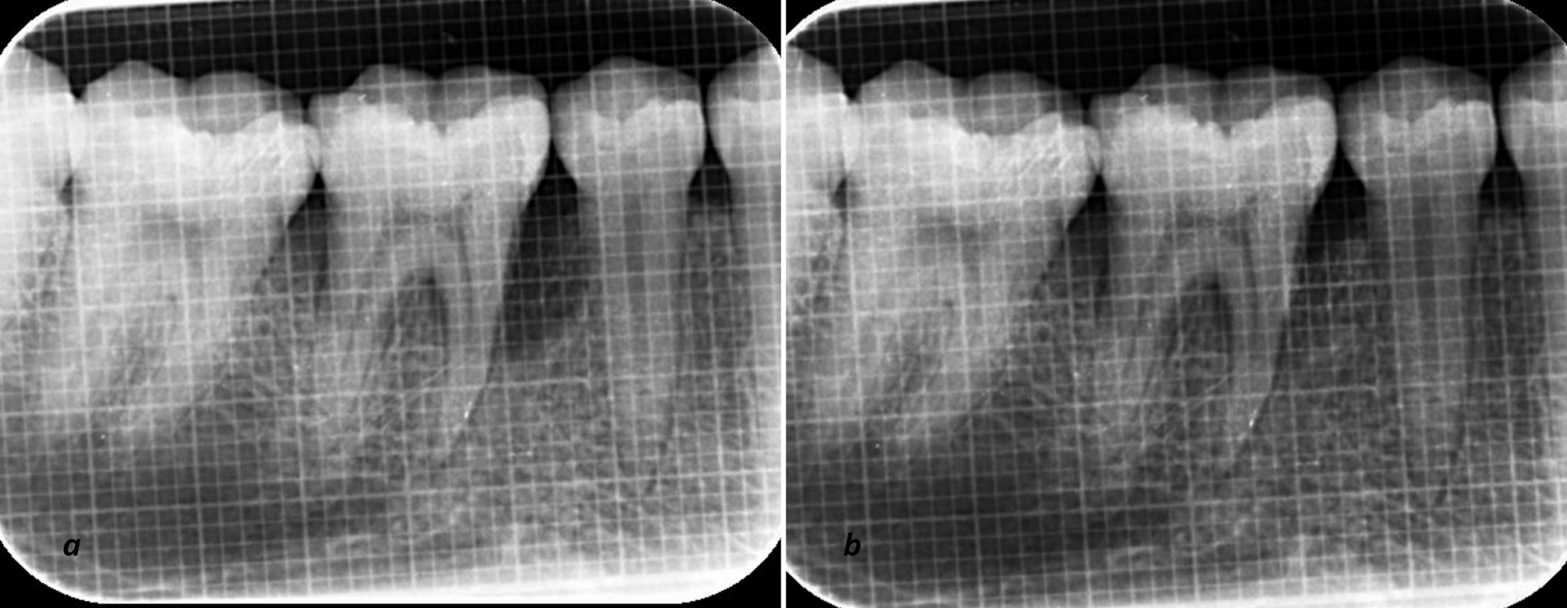
The radiographic (IOPA) evaluation at baseline (a) and after 9 months (b) in the T-PRF group.

Uniformity regarding radiographs was obtained with a long-cone paralleling technique with Rinn’s film holder and a radiographic grid of 1×1-mm framework (Figures 3 and 4). An effort was made to take pre- and postoperative radiographs at a similar projection geometry and optical density. The images were scanned, and measurements, such as cementoenamel junction (CEJ) to the base of the defect (BOD) for defect fill and alveolar crest (AC) to BOD for resolution of defects, were evaluated at baseline and nine months postoperatively based on Meffert et al,^21^ and using ImageJ software (Wayne Rasband, National Institute of Health, USA).

**Figure 5:**
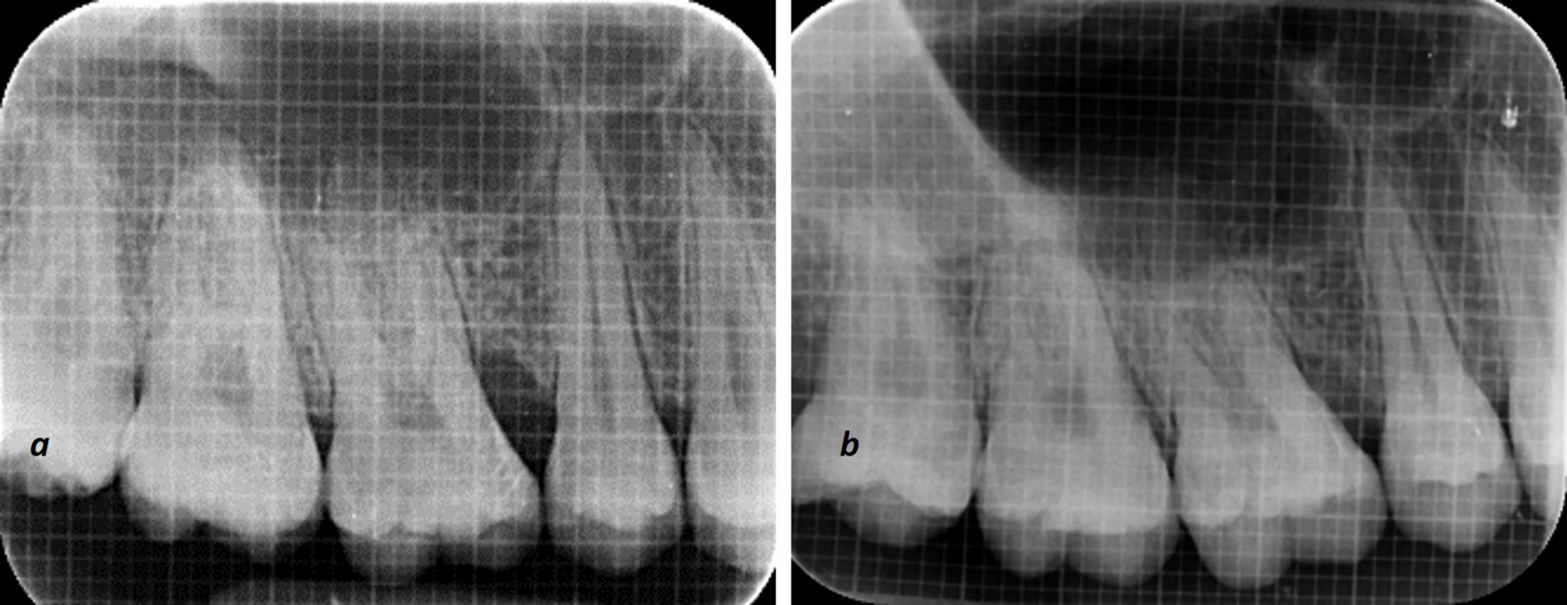
The radiographic (IOPA) evaluation at baseline (a) and after 9 months (b) in the L-PRF group.

### Primary and secondary outcomes 

The percentages of bone fill and original defect resolution were the primary outcomes, whereas PPD, CAL, GI, and PI were secondary outcomes of the study.

### Presurgical therapy 

After thorough phase 1 therapy, hygiene maintenance was reinforced for the patients. Re-evaluation was carried out after eight weeks,^22,23^ and patients were recruited to the surgical phase after checking the sites’ suitability. Blood tests were performed beforehand, and the surgery was planned. Both groups underwent a similar surgical technique.

### Preparation of T-PRF and L-PRF clots 

T-PRF and L-PRF clots were prepared 20 minutes before surgery based on Tunali et al^12^ (3500 rpm for 15 min) and Choukroun et al^7^ (2800 rpm for 12 min) protocols. 10 mL of blood was drawn from the antecubital vein and transferred into sterile medical-grade titanium tubes (Supra Alloys, Camarillo, California, USA) and sterile glass tubes, respectively. Centrifugation (REMI R-8C) was carried out, and due to the difference in densities, three layers were formed. The top layer was pipetted out with a sterile dropper, which consisted of acellular plasma; the bottom layer was the red cell layer, and the middle layer consisted of T- & L-PRF clots. These T- & L-PRF clots were retrieved from titanium and glass tubes using a sterile tweezer and compressed under gauze to form T-PRF & L-PRF membranes, respectively. These membranes were cut into two equal parts, and one part was used for filling the defect site, and the other part was placed over it as a membrane (Figure 2).

### Surgical procedure 

Regarding the surgical procedure (Kirkland’s modified flap operation was performed in both groups), after achieving profound anesthesia with 2% lignocaine hydrochloride with 1:80,000 adrenaline, crevicular and interdental incisions were given using a #15/12 blade. Both the buccal and lingual flaps were reflected to expose the IBD. Thorough debridement was carried out, and the membranes (L-PRF & T-PRF) were placed at the defect sites as a filling material, and as a membrane over the defect. The flaps were approximated using 3-0 non-resorbable silk sutures (Mersilk-Ethicon, Jhonson and Jhonson Ltd, India) with simple interrupted suturing technique. The surgical site was covered with a periodontal pack, and the sutures were removed after ten days.

### Postsurgical phase

Postoperative instructions were given. Amoxicillin (500 mg tid) diclofenac sodium and paracetamol tablets (tid) for five days and 0.2% chlorhexidine gluconate (Rexidin^TM^) rinses were also prescribed twice daily for 14 days. The patients were asked to refrain from brushing the surgical site for at least for 10 days. Then, the patients were maintained under a strict maintenance program and recalled at regular weekly intervals up to one month and then once a month up to nine months.

### Statistical analysis 

The data were tabulated and subjected to analysis using SPSS 22 (SPSS, IBM, Chicago, IL, USA). Paired and unpaired t-tests were used for intra- and inter-group comparisons. Values of PPD, CAL, GI, and PI were expressed as means and standard deviations at baseline and after 3, 6, and 9 months. Even defect fill and defect resolution were expressed in mean percentage changes at baseline and nine months.

## Results

Twenty-six patients, consisting of 11 patients with a mean age of 29.5 years in the T-PRF group and 15 patients with a mean age of 36.1 years in the L-PRF group, underwent surgical therapy. Gender distribution was about six males and five females in the T-PRF group, with nine males and six females in the L-PRF group (Table 1).

**Table 1:** Demographic data showing age, gender, and osseous defects

**Characteristics**	**T-PRF**	**L-PRF**	**Total**
**Total subjects**	11	15	26
**Mean age (in years)**	29.5	36.1	
**Males**	6	9	15
**Females**	5	6	11
**Teeth treated**	Molars	Molars	
**Mandibular**	12	13	
**Maxillary**	5	4	
**Total osseous defects**	17	17	34

GI and PI values in an intra-group comparison between T-PRF and L-PRF showed a statistically significant reduction from baseline to 3- and 6-month intervals. However, in the inter-group comparison, there were no significant differences between the time intervals (Table 2). PPD and CAL values in both the T-PRF and L-PRF groups showed statistically significant improvements in the intra-group comparisons at different time intervals, whereas the results were non-significant in inter-group comparisons (Table 2). Pocket depths, CAL, GI, and PI did not show any significant differences between the T-PRF and L-PRF groups from baseline to 9 months postoperatively (Table 3).

**Table 2:** Comparison of gingival index, plaque index, probing pocket depths, and clinical attachment level improvements from baseline to 9 months in both study groups

**Time interval**	**Gingival index**		
	**T-PRF (n=17)**	**L-PRF (n=17)**	**P-value**
**Baseline**	1.71±0.44	1.46±0.48	0.134 (NS)
**9 months**	0.26±0.14	0.30±0.16	0.467 (NS)
**P-value**	<0.001*	<0.001*	
**Plaque index**			
**Baseline**	1.74±0.34	1.79±0.5	0.721 (NS)
**9 months**	0.45±0.36	0.71±0.38	0.051 (NS)
**P-value**	<0.001*	<0.001*	
**Probing pocket depths**			
	T-PRF	L-PRF	p-value
**Baseline**	8.65±1.50	8.18±1.29	0.333 (NS)
**9 months**	4.82±1.78	3.29±1.1	0.740(NS)
**P-value**	<0.001*	<0.001*	
**Clinical attachment level**			
**Baseline**	9.06±1.56	8.59±1.33	0.350 (NS)
**9 months**	4.12±1.58	4.35±1.46	0.654 (NS)
**P-value**	<0.001*	<0.001*	

(*statistical significance; NS: Non-significant; n: sample size)

**Table 3:** Comparison of mean differences from baseline to 9 months for various clinical parameters in the T-PRF and L-PRF groups

**Clinical parameters and groups (n=17)**		**Mean ± SD**	**P-value**
**Pocket depths**	**T-PRF**	5.24±1.30	0.369 (NS)
	**L-PRF**	4.88±0.93	
**Clinical attachment level**	**T-PRF**	4.94±1.43	0.136 (NS)
	**L-PRF**	4.24±1.25	
**Gingival index**	**T-PRF**	1.45±0.43	0.109 (NS)
	**L-PRF**	1.17±0.55	
**Plaque index**	**T-PRF**	1.29±0.51	0.344 (NS)
	**L-PRF**	1.08±0.72	

(NS: Non-significant, n: sample size)

Regarding radiographic parameters, for original defect fill (CEJ to BOD) and original defect resolution (AC to BOD), intra-group comparisons revealed a statistically significant difference (P<0.001) in both the T-PRF and L-PRF groups when baseline parameters were compared to 9 months. However, in terms of mean percentage defect fill, T-PRF showed a significantly higher defect fill when compared to L-PRF at 9 months (P<0.004) Furthermore, there was a non-significant difference (P=0.096) in percentage defect resolution between T-PRF and L-PRF groups after 9 months (Table 4).

**Table 4:** The mean percentages of defect fill and defect resolution in both study groups

**Study groups (n=17)**	**Baseline**	**9 months**	**P-value**	**%**
**Mean defect fill**				
**T-PRF**	5.52±2.97	3.47±1.61	<0.001*	62.28±21.36
**L-PRF**	6.97±3.68	5.29±2.63	<0.001*	36.21±21.71
**P-value**	0.214 (NS)	0.020*		<0.004**
**Mean defect resolution**				
**T-PRF**	3.14±1.65	1.81±0.72	<0.001*	41.55±19.23
**L-PRF (n=17)**	4.70±2.36	3.18±1.68	<0.001*	30.96±16.75
**P-value**	0.032*	0.004*		0.096 (NS)

(*statistical significance; NS: Non-significant)

## Discussion

PRF was successful for the restoration of lost periodontal tissues after stimulating the adjacent cells to release growth factors at constant time intervals.^24^ Since L-PRF has previously shown promising results, the present study considered it as the standard (control) and compared it with T-PRF. T-PRF, too, is an autologous platelet concentrate where titanium activates platelets, resulting in a thicker fibrin membrane. Due to the limited number of studies reporting the use of T-PRF, it was used in the present study as a novel biomaterial in the treatment of periodontal intra-bony defects. All the sites that were treated in the present study were closed with 3-0 silk sutures because it was readily available, inexpensive, and easy to use, with excellent tensile strength. Postoperatively, chlorhexidine gluconate mouthwash was used, which might have also helped decrease plaque accumulation, resolving gingival inflammation.^25^ Even previous studies^26,27^ used this suture material for suturing and achieved proper postoperative healing without complications.

The present study showed a statistically significant reduction in PI and GI values from the initial visit to 9 months. However, inter-group comparisons revealed a non-significant result, indicating that the change was the same in both T-PRF and L-PRF groups. This is consistent with Chatterjee et al,^19^ Chacko et al,^28^ and Mitra, DK et al.,^29^ who showed a significant decrease in PI and GI values during a 9-month follow-up period for intra-group comparisons and a non-significant reduction in inter-group comparisons. This can be interpreted as useful; repeated removal of plaque biofilm as well as oral hygiene maintenance by the patients contributed to a proper environment for healing and decreasing gingival inflammation. Thus, it is expected that wound healing occurs uneventfully only when patients maintain good oral hygiene.^30^

In the present study, intra-group comparisons showed a significant decrease in PPD from baseline to 9 months in both the T-PRF and L-PRF groups, whereas inter-group comparisons revealed non-significant differences. This was consistent with Chatterjee et al,^19^ Sharma and Pradeep^8^, and Patel et al.^31^ However, the mean reduction in PPD values of the L-PRF group in the present study was higher than that in a study by Patel et al,^31^ where the follow-up period was one year. Thus, PRF (both T-PRF and L-PRF) serves as a reservoir for growth factors and cytokines, which decrease the levels of matrix metalloproteinase-8 and interleukin-1β and increase tissue inhibitors of matrix metalloproteinase-1 levels, resulting in periodontal soft tissue healing.^32^ Moreover, PRF also helps prevent the migration of epithelial cells.^33^

In the present study, a significant mean CAL gain was noted from baseline to 9 months in both groups in intra-group comparisons, consistent with Chatterjee et al,^19^ and Mitra et al.^29^ This gain in CAL reflects proper wound healing in the presence of T-PRF and L-PRF. Thus, CAL is considered as an endpoint of regenerative attempts around natural dentition in regenerative studies.^34,35^ Even a systematic review conducted by Trombelli et al^3^ concluded that specific biomaterials, such as bone grafts, GTR, growth factors, and platelet concentrates, improved the CAL levels in intra-bony defects. However, in the present study, L-PRF showed a moderate CAL gain, which was much higher than that reported by Patel et al.,^31^ who carried out a 12-month postoperative follow-up. This might be due to a higher clinical attachment loss recorded at baseline, resulting in a better gain in CAL values during the follow-up period in the present study

Concerning radiographic parameters, bone changes were assessed according to Meffert et al,^21^ and image analysis was completed using ImageJ software. This way, human errors are eliminated to a large extent compared to manual counting, and digitizing the unit (images) yields precise values. Even an in vitro study concluded that ImageJ software could be considered an accurate method for assessing bone changes.^36^ In the present study, three-walled IBDs were treated as the number of bony walls shows the effectiveness of stabilizing a biomaterial, blood clots, and formation of new blood vessels between the periodontal ligament and bony walls.

In the present study, the intra-group comparison revealed a significant original defect fill in both the T-PRF and L-PRF groups. In contrast, inter-group comparisons significantly favored the T-PRF group, which is quite different from the results reported by Chatterjee et al,^19^ who found a higher percentage of defect fill for T-PRF with no significant difference. Moreover, the results of inter-group comparisons concerning original defect fill are different from the results reported by Mitra et al,^29^ who showed no significant difference between the T-PRF and L-PRF groups.

In the present study, the L-PRF group showed similar results to studies conducted by Patel et al,^31^ and Thorat et al,^22^ where there was a significant defect fill along with decreased PI, reduced PPD, and gain in CAL at 12-month postoperative follow-up. Pradeep et al^9^ concluded that L-PRF placement in alveolar defects improved the clinical and radiographic parameters. This could probably be due to variations in selection norms of samples, such as defect depths and defect angle. Apart from this, PRF promotes the osteoprotegerin levels which stimulate bone formation through osteoblastogenesis.^37^

In the present study, the percentages of defect resolution in the T-PRF group were higher than those in the L-PRF group, with no significant difference. In contrast, Mitra et al^29^ showed less depth reduction in the T-PRF group, which might be due to a small sample size, or differences in defect morphology that play a role in resolution. These improvements in radiographic parameters in the present study might be because longer resorption time of T-PRF stimulated more cells, which in turn increased osteoprotegerin levels, and new bone was formed through osteoblastogenesis.^37^

The results of the present study regarding a decreased PI, a reduction in PPD, a gain in CAL, and an improvement in bone fill nine months postoperatively were consistent with a recent study by Ustaouglu et al.^38^ They achieved a significant reduction in PPD, gain in CAL, and improvement in defect depth nine months postoperatively in their T-PRF, GTR (guided tissue regeneration), and OFD study groups. However, they reported no significant difference between the T-PRF and GTR groups. Besides, there was a greater improvement in clinical and radiographic parameters postoperatively in the T-PRF and GTR groups when compared to OFD alone. They concluded that the application of T-PRF and GTR was much better in improving clinical and radiographic parameters than OFD alone. Tunali et al^12^ reported that T-PRF induced new bone formation in a rabbit model with improvements in various clinical and radiographic parameters. This might be due to thicker fibrin meshwork with higher cellular entrapment, resulting in adequate growth factor release, which in turn stimulate a higher number of cells for new bone formation.^14,37^

The limitations of the present study might be a small sample size, lack of histological analysis of the formed tissue, open flap debridement not taken as control, lack of the use of advanced radiographic techniques due to financial constraints, higher cost of titanium tubes, and short follow-up time.

## Conclusion

Under the limitations of the present study, the present study showed that both T-PRF and L-PRF improved the clinical parameters, indicating that they are useful in soft tissue healing. Due to more bone fill percentage by stimulating the osseous cells, T-PRF helped significantly in hard tissue healing compared to L-PRF.

## Authors’ Contribution

The authors contributed in the following way. GSS was responsible for the concept or design of the work, data acquisition or analysis, interpretation, and drafting of the work. HB was responsible for the concept or design, revising, and final drafting of work. MA was responsible for drafting and revising the work. CS was responsible for the analysis and revision of the work.

## Funding

The present study was self-funded by the authors themselves, and no funding was received from any agency.

## Competing Interests

The authors declare no competing interests with regards to the authorship and/or publication of this article.

## Ethics Approval

The study was approved by the institutional review board under the code IDS/ETHCC/17/08.
